# Insulin resistance in prostate cancer patients and predisposing them to acute ischemic heart disease

**DOI:** 10.1042/BSR20182313

**Published:** 2019-07-29

**Authors:** Udayan Ray, Sarbashri Bank, Madawa W. Jayawardana, Jahar Bhowmik, Frank Redwig, Pradipta Jana, Suman Bhattacharya, Emili Manna, Subrata Kumar De, Smarajit Maiti, Philip Roberts-Thomson, Venkat Parameswaran, Asru K. Sinha

**Affiliations:** 1 Department of Pathology, The Royal Hobart Hospital, Tasmania, Australia; 2 Department of Biochemistry, Sinha Institute of Medical Science and Technology, Kolkata, India; 3 Deptartment of Biochemistry, OIST, Vidyasagar University, Medinipur, West Bengal, India; 4 Department of Statistics, Data Science and Epidemiology, Swinburne University of Technology, Melbourne, Australia; 5 Department of Botany, University of Calcutta, West Bengal, Kolkota, India; 6 Centre for Life Sciences, Vidyasagar University, Medinipur, West Bengal, India; 7 Department of Zoology, Vidyasagar University, Medinipur, West Bengal, India; 8 Department of Pharmacology and Toxicology, Higuchi Biosciences Center, University of Kansas, Lawrence, Kansas 66047, U.S.A.

**Keywords:** dermcidin, insulin resistance, prostate cancer, nitric oxide, HOMA score, hs-cTropononT

## Abstract

Lack of insulin or insulin resistance (IR) plays a central role in diabetes mellitus and makes diabetics prone to acute ischemic heart disease (AIHD). It has likewise been found that many cancer patients, including prostate cancer patients die of AIHD. Previously it has been delineated from our laboratory that dermcidin could induce anomalous platelet aggregation in AIHD and also impaired nitric oxide and insulin activity and furthermore dermcidin was also found in a few types of cancer patients. To determine the role of this protein in prostatic malignancy, a retrospective case–control study was conducted and blood was collected from prostate cancer patients and healthy normal volunteers. So, we measured the level of dermcidin protein and analyzed the IR by Homeostasis Model Assessment (HOMA) score calculation. Nitric oxide was measured by methemoglobin method. HDL, glycated hemoglobin (HbA1c), BMI, hs-cTroponin-T were measured for the validation of the patients’ status in the presence of Dermcidin isoform-2 (DCN-2). Multiple logistic regression model adjusted for age and BMI identified that the HOMA score was significantly elevated in prostate cancer patients (OR = 7.19, *P*<0.001). Prostate cancer patients are associated with lower level of NO and higher level of both proteins dermcidin (OR = 1.12, *P*<0.001) and hs-TroponinT (OR = 1.76, *P*<0.001). From the results, it can be interpreted that IR plays a key role in the pathophysiology of prostate cancer where dermcidin was the cause of IR through NO inhibition leading to AIHD was also explained by high-sensitive fifth generation cTroponin-T (hs-cTroponinT) and HbA1c level which are associated with endothelial dysfunction.

## Introduction

Malignant neoplasm is one of the leading causes of mortality across the world, both in developing and the developed nations. Approximately 9.6 million deaths have been found to occur with cancer in 2018 according to the World Health Organization and approximately 1.8 million of them are prostate cancer [1–3]. Prostate cancer is the fourth leading cause of cancer deaths in men in the world [1–3] and it is the most common cancer for men over 55 years of age [4]. In Australia, there is a chance of one in five men developing prostate cancer before the age of 55 [4]. In North America, this figure is approximately one in seven men [5]. There are many epidemiological evidence-based studies in the literature indicating that prostate cancer is inherited in many cases [4,6]. It is an already known fact that the BRCA1 and BRCA2 genes which are involved in breast cancer [7], ovarian cancer [8] and pancreatic cancer [6] have a pivotal role in the growth of prostate cancer [5,9–11] as well. However, the mechanism behind the problem remains obscure in that area. It has been found that 30% prostate cancer at the age of 50 and 80% are at the age of 70, so it is well known that prostate cancer is the old-aged disease. It has been also identified that many of the prostate cancer patients die from cardiovascular diseases [12,13]. It has been reported that the risk of coronary artery disease (CAD) is increased during first 6 months after prostate cancer diagnosis, and metastasis is associated with an increased risk of CAD [14]. Hypertension, diabetes mellitus, chronic kidney diseases and malignancy play the central roles in the pathophysiology of acute ischemic heart disease (AIHD) in these cardinal non-communicable diseases in the modern civilization [15–17]. In prostatic malignancy, patients die of AIHD, urinary tract infection, chronic kidney failure and cerebrovascular stroke. Metabolic syndromes are the risk factors of the prostate cancer [18] but the mechanism of insulin resistance (IR), elevated insulin-like growth factors and even hyperinsulinemia have not been fully elucidated in the setting of prostate cancer.

CAD and cancer share common risk factors, such as smoking, and there is a moderately increased risk of tobacco-related cancers among survivors of myocardial infarction (MI). CAD may predate the development of cancer from the common pathophysiology of IR or may result from treatment of cancer itself. We reported from our laboratory that insulin is an antithrombotic humoral factor for the prevention of CAD through the production of nitric oxide [19]. Therefore, it could be speculated that IR might be the important issue in prostate cancer through the suppression of nitric oxide synthesis. We have likewise reported that the production of nitric oxide is greatly perturbed in cancer patients [20]. Dermcidin isoform-2 (DCN-2), an 11-kDa small, environmentally stress-induced protein, plays the main role in IR and produces atherosclerosis in the pericardial arteries of the heart and as a result cardiac cell death and acute MI (AMI) develop [21,22]. DCN-2 protein functions as an inhibitor of all forms of nitric oxide synthases and inhibits the action of insulin in AIHD [21]. If there is an acute myocardial injury or necrosis, there is sharp elevation in hs-TroponinT level in the circulation of the patients. In the present study, we looked into the basic biochemical phenomenon which takes on an important pathophysiologic part at the molecular level to precipitate the AIHD in prostate cancer patients. Cardiovascular disease was found to occur in many cancer patients and here we considered only the prostate cancer patients to observe the IR by Homeostasis Model Assessment (HOMA) analysis. With the elevated IR condition, we also found some other increased level of risk factors of AIHD in prostate malignancy. And we found here dermcidin protein, which contributes one of the central roles in the regulation of other risk factors of vascular diseases. So, in this clinical experimentation, we have unveiled that these factors can control AIHD in prostate cancer wherever chemo, radiation or hormonal therapy-induced carcinogenic shock/dysfunction would be expected. Our objective was to evaluate if there is any correlation between IR (through the mediation of dermcidin protein, NO, HOMA score, Insulin, HDL) and status of prostate cancer outcome which is evaluated as health status. We were also intended to observe the relation of NO level with cancer outcome because our primary objective was to verify the cancer state and its influence on cardiac outcome.

## Methods

### Chemicals

Goat anti-rabbit immunoglobin-G alkaline phosphatase was purchased from Sigma–Aldrich. Enzyme-linked immunosorbent assay (ELISA) plates were from Nunc Roskilde, Denmark. Dermcidin primary antibody was from Abcam. High-sensitive fifth generation cTroponin-T (hs-cTroponinT). All the chemicals were of analytical grade.

### Selection of prostate cancer patients

The present study was based on a subset of patients drawn from a retrospective case–control study of prostate cancer patients who were seen at the Royal Hobart Hospital, Tasmania, Australia and Sinha Institute, India. Patients were eligible for the study if they met all of the following criteria: age > 40 years; have not taken any aspirin-like medicine at least 1 month before the blood withdrawal. Those patients were included who were initially diagnosed with cancer and before the commencement of their formal treatment/medication blood sample was collected. We were able to avoid the influence of other disease and any medications. So, no cancer medicine was taken by the patients previously and before starting the cancer treatment, blood sample was withdrawn from the prostate cancer patients. Patients were excluded if any of the following criteria were encountered: diabetics and on insulin, nitrates and non-steroidal anti-inflammatory medications (NSAIDs), suffering from any life-threatening diseases; heart-failure and taking different kinds of medicines. A total of 27 prostate cancer patients (*n*=27) was considered and a total of 25 healthy control subjects who were free from any type of disease complicacy (subjects had no high blood pressure and had normal lipid profiles) and were not taking any kind of medicine since last 1 month, were included in the study.

### Diagnosis of prostate cancer

All the suspected prostate cancer patients were diagnosed for its confirmation. The cancer patients who had PSA levels > 10 ng/ml were included in the experiment. Prostate cancer was confirmed by biopsy of prostate tissue (here it is noted that biopsy was not done in our experiment, but for the treatment of the cancer patients, we just took the data), both pre-operatively and post-operatively were taken into consideration and noted [Gleason score was (4+3), i.e., grade-3] otherwise doubtful samples were excluded from the study.

### Collection of blood

A total of 1.5 ml of blood was collected by vein puncture in citrate solution (1 vol. of citrate solution: 9 vol. of blood sample) from the study participants, including both prostate cancer patients (here it was noted that blood was drawn from the patients to know the parameters of the sample for their treatment purpose, we took a little amount of blood from the withdrawal sample) and healthy control subjects [23].

### Preparation of plasma

Plasma was prepared from whole blood of study participants by centrifuging at 5000 rpm for 12 min as described previously [23].

### ELISA for dermcidin

Plasma level of dermcidin was quantified by ELISA by using dermcidin antibody as described before [24].

### Assay of nitric oxide

Production of nitric oxide was measured by the methemoglobin method by using Beckman spectrophotometer model DU by the spectral changes (575–630 nm) as described in [25,26] and the NO assay was confirmed by Chemiluminescence method [27].

### HOMA score analysis

For the estimation of IR, HOMA-estimated IR (HOMA-IR) calculation was used for the study. In prostate cancer patients, HOMA score was analyzed by glucose level and insulin level. HOMA estimates the β-cell function and insulin sensitivity [28]. It was analyzed by means of the multiplication of the fasting plasma glucose (FPG) by fasting plasma insulin (FPI) and then divided by the constant 22.5 (the resultant value below 2.5 is normal). Insulin was measured by the Chemiluminescence assay in Immulite system and glucose was measured by Abbott Architect c-8000 by using the hexokinase method.

### HDL measurement

The plasma HDL level of the study participants was measured by using Architect ci8000/Cobas-6000 through immunoturbidimetric assay method.

### hs-cTroponinT measurement

Cardiac troponin-T is used for the diagnosis of AMI. hs-cTroponinT is able to measure very low level of troponin in AMI and was used here for the diagnostic accuracy of AMI. hs-cTroponinT was measured by Cobas 6000 Immuno-analyser.

### Glycated hemoglobin measurement

Bio-Rad’s mini-column D-10 equipment using high performance liquid chromatography (HPLC) ion exchanger with mobile phase (gradient) and spectrophotometric detection was used to obtain glycated hemoglobin (HbA_1c_) measurements.

### Statistical analysis

Both descriptive and inferential statistical analyses were applied to the collected data using the R statistical software [29]. Continuous variables were expressed by the mean value and the corresponding 25th and 75th percentiles. The Student’s *t* test was used for comparison of continuous variables. Multiple binary logistic regression model was applied to assess the association of the HOMA score, dermcidin, NO levels and hs-cTroponinT with the prostate cancer status, adjusting for the demographic variable age and the clinical variable BMI. A bias-reduction method that was proposed in [30] was considered when fitting the logistic regression models using the R software package *brglm* [31]. Bias-reduced methods are well-suited for small sample studies, which will have lower standard errors for the estimated parameters as compared with the traditional maximum likelihood estimates and it accounts for the complete or quasi separation issues in logistic regression [30,32]. A two-tailed *P*-value of less than 0.05 was considered to indicate a statistically significant difference for all the analyses performed. All statistical analysis was performed using R statistical software [33].

## Results

In Table 1, we summarize the characteristics of the two groups of subjects in the study. We noticed a highly significant difference between prostate cancer patients and control group in demographic and clinical characteristics. Patients’ group had a higher level of results compared with the control group in all the characteristics, except for HDL and NO.

**Table 1:** Characteristics of the study population

Characteristics	Case (*n*=27)^1^	Control (*n*=25)^1^	*P*-value^2^ (95% C.I.)
Age (years)	70 (66–77)	58.7 (55–65)	<0.001 (6.4, 16.3)
BMI (kg/m^2^)	27.4 (26.5–28)	26.2 (25–26.8)	0.001 (0.5, 1.9)
HbA_1c_	6.2 (6.2–6.5)	5.4 (5.3–5.5)	<0.001 (0.8, 1.0)
HOMA score	5.2 (4.2–6.2)	2.3 (2.0–2.3)	<0.001 (2.2, 3.5)
Glucose	5.7 (4.9–6.5)	4.8 (4.4–5.2)	<0.001 (0.4, 1.4)
Insulin	21.8 (19.7–24.0)	10.4 (9.5–11.5)	<0.001 (9.4, 13.5)
HDL	0.9 (0.8–1.0)	1.3 (1.2–1.4)	<0.001 (−0.5, −0.3)
Dermcidin (nM)	71.5 (59.8–82.4)	17.9 (8.5–27.8)	<0.001 (45.9, 61.2)
NO (nmol/10^8^ platelets/ml)	0.2 (0.1–0.3)	1.4 (1.3–1.7)	<0.001 (−1.4, −1.1)
hs-cTroponinT	20.4 (14.8–21.5)	4.9 (3.4–5.9)	<0.001 (12.3, 18.7)

1 All the values are medians with 25th and 75th percentiles in parentheses.

2 *P*-values were determined from the Student’s *t* test.

### Dermcidin level in prostate cancer patients and corresponding nitric oxide level

As most of the prostate cancer patients die because of cardiac diseases, so we wanted to detect the DCN-2 protein and nitric oxide if any, in prostate cancer patients because NO was also reported to involve with DCN-2 protein in cardiovascular diseases [21,22]. It was found from the ELISA experiment that the average dermcidin level in prostate cancer patients was 71.5 (59.8–82.4) nM. For the healthy control group, the average dermcidin level was 17.9 (8.5–27.8) nM. Binary logistic regression analysis of the dermcidin against the prostate cancer status (reference group: healthy participants) shows that there is a positive association with dermcidin and the prostate cancer status (Table 2). Therefore, on average, dermcidin level is higher in prostate cancer patients as compared with the healthy participants of the study.

**Table 2:** Bias-reduced logistic regression results after adjusting for age and BMI

Characteristics	Adjusted odds ratio^1^	*P*-value
Dermcidin (nM)	1.12 (1.04, 1.21)	0.003
NO (nmol/10^8^ platelets/ml)	0.02 (1.35E-03, 1.85E-01)	<0.001
HOMA score	7.19 (2.25, 22.94)	<0.001
hs-cTroponinT	1.76 (1.18, 2.60)	0.005

1 Odds ratios are adjusted for the demographic variable age and clinical variable BMI. 95% confidence intervals are given in parentheses.

In contrast with a weak positive correlation with the healthy control group, we observed a moderate negative correlation between NO and dermcidin levels in prostate cancer patients (Table 3). Hence, on an average the level of NO in prostate cancer patients is lower than the healthy participants’ of the study. The average production of NO in prostate cancer patients was 0.2 (0.1–0.3)^1^ nmol/10^8^ platelets/ml. However, the average production of NO in healthy group was 1.4 (1.3–1.7)^1^ nmol/10^8^ platelets/ml. Binary logistic regression model (adjusting for age and BMI) indicated that there is a negative association with NO and the prostate cancer status.

**Table 3:** Pearson’s correlation coefficient values for the prostate cancer patients group and the healthy control group (in parentheses)

	Age	BMI	HbA1c	HOMA	Glucose	Insulin	HDL	Dermcidin	NO	hs-cTroponinT
Age	1	0.020 (−0.105)	0.097 (0.298)	0.156 (−0.134)	−0.002 (−0.061)	0.398^1^ (0.129)	−0.395^1^ (0.289)	−0.090 (0.457^1^)	−0.022 (0.149)	0.193 (0.041)
BMI		1	0.136 (−0.084)	−0.327 (0.172)	0.066 (−0.306)	−0.306 (0.069)	−0.189 (0.017)	−0.224 (−0.288)	−0.084 (−0.001)	−0.055 (−0.018)
HbA_1c_			1	0.211 (0.253)	0.521^2^ (0.269)	0.010 (0.105)	−0.196 (0.266)	0.178 (0.390)	−0.043 (−0.032)	0.217 (0.054)
HOMA				1	0.331 (−0.178)	0.791^2^ (0.347)	−0.157 (0.199)	0.287 (0.070)	0.008 (0.047)	−0.244 (0.030)
Glucose					1	0.002 (−0.786^2^)	−0.006 (−0.243)	0.163 (0.222)	0.107 (−0.073)	−0.153 (0.069)
Insulin						1	−0.272 (0.249)	0.361 (0.037)	−0.095 (0.103)	−0.038 (0.038)
HDL							1	−0.277 (0.402^1^)	0.101 (0.294)	−0.285 (0.125)
Dermcidin								1	−0.503^1^ (0.187)	0.054 (0.005)
NO									1	−0.148 (0.176)
hs-cTroponinT										1

1 Significance at 5% level.

2 Significance at 1% level.

### Determination of HOMA-IR score analysis in prostate cancer patients and simultaneous detection of hs-cTroponin level

From the epidemiological studies, it has been reported that IR is found in malignancy [34] and as IR is well known in cardiac disease, so troponinT might be elevated. So, to test the hypothesis of IR in prostate cancer HOMA-IR was performed. In general, HOMA was found to increase in the prostate cancer patients, which indicates IR in prostate cancer patients (Table 1). The average HOMA score for the prostate cancer patients was 5.2 (4.2–6.2)^2^ and for the healthy control group it was 2.3 (2.0–2.3)^2^. There was a significant difference (*P*-value <0.01) in HOMA score values between the two groups (Table 1). Furthermore, the binary logistic regression model (adjusted for age and BMI) confirmed that there is a significant positive association with the HOMA score and the prostate cancer status (Table 2).

hs-cTroponinT is an important marker protein to determine the level of AMI patients [35,36]. In the present study, it was found that this marker protein amplified in prostate cancer patients with the simultaneous increase in HOMA score levels. There was a significant difference (*P*<0.001) in hs-cTroponinT levels across the healthy and the cancer group of patients (Table 1), where the level of hs-cTroponinT is higher in prostate cancer patients as compared with the healthy group. Furthermore, the logistic regression model after adjusting for the age and BMI suggested there is a positive association with hs-cTroponinT and the prostate cancer status.

## Discussion

Herein it has been found that the risk factors of cardiovascular diseases are prevalent in the prostate malignancy due to IR which has been explained by HOMA score analysis and diminution of nitric oxide synthesis. From our previous experiment, we found that the death rate of the cancer patients is increasing due to AMI where NO is crucially involved [20]. The plasma level of NO, that has been reported to possess various anti-neoplastic properties [37–39], was found to be diminished due to the impairment of insulin-activated nitric oxide synthase (IANOS) as a result of the appearance of a novel antibody against the enzyme in the circulation in various cancers compared with normal control [37]. It was reported that the resumption of NO synthesis through the neutralization of antibody resulted in favorable modifications of various cancer-associated pathophysiologic consequences [37]. We have reported before that restoration of nitric oxide in physiological level might be helpful to prevent the MI death *in vitro* [21].

In some instances, it has been reported that higher degree of testosterone somehow might be the causative agent in prostate malignancy, though the exact mechanism was speculative [40]. From our previous experiment, we reported that in the presence of testosterone (male androgen hormone), aggregation of platelets was higher compared with control, whereas only ADP-induced platelet aggregation is a normal phenomenon [41]; so, prostate cancer patients are prone to AIHD in such cases. Most of the AIHD or CAD patients would have an IR and nitric oxide synthesis anomaly in their system, which ultimately results in a failed fight against inflammatory and ischemic events. Eventually the situation arises where platelets aggregate in the coronary arteries or cerebral arteries culminating an AMI or cerebrovascular stroke, respectively. It has been reported that dermcidin is a cancer cachectic and proteolysis inducing factor (PIF) [42]; it was also found to overexpress in malignant proliferative cells [43]. This stress-induced protein, which was found in the circulation of AMI and ACS patients, was the platelet aggregator and the inhibitor of the aspirin effect through the impairment of nitric oxide [21,22]. We reported from our laboratory that dermcidin-induced hyperglycemia was due to the impairment of NO [44,45] which causes endothelial dysfunction relating to the elevated HbA1c [46,47]. Insulin, which is an anti-thrombotic factor, plays a very important role in the inhibition of AIHD [19,48] through the production of nitric oxide [49] by the expression of eNOS gene [50]. Insulin shows its thrombolytic activity by synthesizing plasmin from plasminogen and helped in the breakage of fibrinogen bond [51]. Insulin also imparts in the skeletal muscle vasodilation through the release of nitric oxide [52], as such nitric oxide is the second messenger of insulin [53]. In different types of cancer, the production of nitric oxide was found to be zero or significantly diminished [20] and it was also found that the low level of nitric oxide was the responsible factor for the creation of acute ischemic cardiac pain, so nitro compounds (nitroglycerin, isosorbide dinitrate) when administered can help in the neutralization of this acute chest pain through the expression of nitric oxide regulating protein [54]. So, it can be argued that diminished nitric oxide might be the factor in prostate cancer patients predisposing to AIHD. In case of IR in prostate cancer patients, heterogeneous binding of insulin was inhibited on the platelet surface and that is why the level of insulin was found to be low and here DCN-2 might impart the major role in the reduction or impairment of insulin action in the condition as described before [21]. It was also reported that DCN-2 was involved in different types of cancer progression and metastasis; expression of dermcidin gene was involved in the development of micro-environment of prostate cancer in hypoxia [55] and consequently it was also demonstrated before that the protein was responsible for the aggregation of platelets and inhibition of the effect of aspirin in AMI patients [21].

In the present study, we observed that the dermcidin concentration was higher in prostate cancer patients with the simultaneous low production of NO, while in healthy participants’ nitric oxide production was found to be normal. Physiological level of nitric oxide is very significant because it acts as a messenger molecule in several cellular functions. Furthermore, HOMA score was found to be elevated in prostate cancer patients. This explains the IR in those patients. We also observed that the hs-TroponinT levels were significantly higher in prostate cancer patients. But the fallacy is that the androgen deprivation therapy (ADT) [56] or radiation/chemotherapy is employed in the prevention of prostate malignancy but these agents can induce cardiotoxicity and many of the prostate cancer patients die due to cardiac diseases not from cancer. So, our experimental approach showed that dermcidin might regulate other parameters which are involved in cardiac diseases and if we aware of DCN-2 regulated IR (HOMA score analysis), NO, HbA_1c_ and cTroponinT level, then our experiment might be helpful for the prostate cancer patients (patients are already in the IR state and prone to AIHD development) to whom different types of cardiotoxic therapy would be exposed, because the timing and dosages of therapeutic agents then would be exposed in a controlled manner and cardiotoxicity might be decreased at least. Not only the abovementioned way of exposure, but also the aspirin therapy might be helpful through the inhibition of DCN-2 activity by aspirin’s low and specific dose [57,21].

Though our experimental sample size is not large, but has an important clinical outcome which is explained above. A larger study with more primary cases is required to further validate the research findings of the present study. Furthermore, the case–control study was not an age-matched study. However, to account for this the demographic variable, age was adjusted in the multiple logistic regression models along with the clinical variable BMI. So, despite the small sample size, it can be assumed from the above found results of clinical experimentation that dermcidin could be one of the responsible factors for the creation of prostate malignancy and concomitantly IR here. Previously, it was found that dermcidin impaired the insulin activity through NO inhibition [22,44] and the found result of elevated HbA1c level and lower level of NO are the indication of endothelial dysfunction which actually precipitates cardiovascular disease. Chest pain is the distinctive feature of AMI and low level of NO might be the causative factor of pain in AMI, and cTroponon is the well known marker of the occurrence of AMI. So, from the results it can be inferred that IR in prostate malignancy plays the critical role by the induction of dermcidin protein and other risk factors, which help in the construction of micro-environment of acute cardiac disease in prostate cancer patients and it can be presumed that various anti-cancer therapies might worsen the situation. We found a highly significant correlation between severity of prostate cancer status and cardiac failure outcome. And in this association the role of dermcidin via dysregulation of NO has been very prominent. The oxidative stress condition initiated due to cancer causing factors was the major determinant of DCD production and NO inhibition. That finally resulted in cardiac anomalies.

## Footnotes

1 The 25th and 75th percentiles are given in the parentheses.

2 The 25th and 75th percentiles are given in the parentheses.

## Abbreviations

ACS: acute coronary syndrome
AIHD: acute ischemic heart disease
AMI: acute myocardial infarction
BMI: Body Mass Index
BRCA: Breast Cancer gene
CAD: coronary artery disease
C.I.: confidence interval
DCN-2: Dermcidin isoform-2
ELISA: enzyme-linked immunosorbent assay
eNOS: endothelial nitric oxide synthase
HbA1C: glycated hemoglobin
HDL: high-density lipoproteins
HOMA: homeostasis model assessment
HOMA-IR: HOMA-estimated insulin resistance
hs-cTroponin T: high-sensitive cardiac troponin T
IR: insulin resistance
MI: myocardial infarction
OR: odds ratio
PSA: Prostate-specific antigen
